# Secure Encapsulation and Publication of Biological Services in the Cloud Computing Environment

**DOI:** 10.1155/2013/170580

**Published:** 2013-09-01

**Authors:** Weizhe Zhang, Xuehui Wang, Bo Lu, Tai-hoon Kim

**Affiliations:** ^1^School of Computer Science and Technology, Harbin Institute of Technology, Harbin 150001, China; ^2^Network and Information Center, Harbin Institute of Technology, Harbin 150001, China; ^3^School of Computer and Information Science, University of Tasmania, Virginia Court, Sandy Bay, Hobart, TAS 7001, Australia

## Abstract

Secure encapsulation and publication for bioinformatics software products based on web service are presented, and the basic function of biological information is realized in the cloud computing environment. In the encapsulation phase, the workflow and function of bioinformatics software are conducted, the encapsulation interfaces are designed, and the runtime interaction between users and computers is simulated. In the publication phase, the execution and management mechanisms and principles of the GRAM components are analyzed. The functions such as remote user job submission and job status query are implemented by using the GRAM components. The services of bioinformatics software are published to remote users. Finally the basic prototype system of the biological cloud is achieved.

## 1. Introduction

In recent decade years, bioinformatics is a leading branch of biological science which deals with the study of methods for storing, retrieving, and analyzing biological data [1]. The rising of bioinformatics software becomes a specialized field. Some bioinformatics software products, such as Blast (database search) [2], Clustalw (multiple sequence match) [3], *phylip* (biological phylogenetic analysis software) [4], and EMBOSS (large sequence analysis) [5], have become an indispensable tool for molecular biology research. Open, public, and common are the main features of bioinformatics software. These software products are usually used in various forms of open source code copyright statement and take the Linux operating system as the main platform. They are mainly developed by scientific research institutions, colleges, universities, and other academic departments freely and provide public use. In the bioinformatics software's directory of GNU Project, there exist 15 kinds of software products, and, in the bioinformatics software's directory of the Open Science project, there exists 51 kinds.

However, the development of the open source software usually aims at solving some specific issues for a particular research field. The academic research is its main purpose, and personal interest is its main driving force. So the program often lacks detail documents and necessary user supports. Installation and configuration of this software will need a certain knowledge and experience of computer programming and system maintenance, which makes this software difficult to be used by most biologists in their research. Even if the user is biological information scientist or even the professional system administrator, it is difficult for them to face many software products with limited documents. In addition, with the rapid increasing of biological information's data scale and the further research for the data, some software will be needed in a research project [6]. The common bioinformatics software, including both free software from academic unit and expensive commercial software, far cannot satisfy the above requirements. For the data format, the same DNA or protein sequences in different databases have different storage format and use different input/output formats in different applications. The user must first be familiar with the conversion between these formats [7]. While, there are hundreds of kinds of sequence analysis software products, if the user is not familiar with their application and use methods, he needs to learn how to use the software and how to analyze the results, which is often wrong as well as time consuming. Although there have been many web-based analytical tools [8], the development of bioinformatics software still cannot get rid of the basic layout which takes the separate calculation method as basis, the individual computer program as the center, and the single calculation results as the goal, and some software's output results are difficult to understand for the biologists.

With the development of the web service in the cloud computing, we gradually pay more attention on this technology which contains a huge processing power. However, for the various bioinformatics products, even if we improve the processing power with the web service technology, we cannot solve the problem that the users need to spend a lot of time and energy to be familiar with different bioinformatics software's use methods or data organization structure. Therefore, it is necessary to provide unified and single software encapsulation for the bioinformatics software. Through the encapsulation, a unified interface and simple operation will be provided; thus the backstage software implementation details can be shielded off, and the users' requirements can be completed correctly and efficiently.

The following sections of this paper are organized as follows: Section 2 introduces the bioinformatics software objects to encapsulation and publication; Section 3 puts forward the outline of the bioinformatics software's encapsulation and publication and implements the encapsulation and publication on both Windows and Linux operating systems; Section 4 conducts functional tests for the bioinformatics software to check the correctness of the encapsulation and publication.

## 2. Example of Bioinformatics Software

This paper chooses three bioinformatics software products as example, namely, gene sequences conversion tools *seqret*, gene sequences ORF search tools *getorf*, and the molecular clock based maximum likelihood estimation tools *proml*. Among them, the two executive programs *seqret* and *getorf* are, respectively, encapsulated on both Windows and Linux operating systems. *Proml* is an application running on the Windows platform, so it is only encapsulated on Windows platform.

### 2.1. Gene Sequences Conversion Tools *Seqret*

*Seqret* is mainly used to transfer the sequence files with different formats. For example, if the data provided by the user needs to be processed by *phylip*'s software encapsulation, but the *phylip*'s software encapsulation only supports the sequence file with *.phy* format, then the transfer of the file sequence's format is necessary. Here, it needs to call *seqret.exe*. Next, the parameters of *seqret* are introduced.

*Seqret* has two main parameters: one parameter is the sign of the input file format and the file name and the other is the sign of the output file format and the file name.

The described file manner is required to correspond with the description format specified in Uniform Sequence Address (USA). The USA formats are described as follows.

-  “*file*”: name of the input file. File is a sequence file with  *.seq* as extension name.
-  “*file:entry*”: combination form of file name and sequence ID.
-  “*format::file*”: combination form of input file's organization format and name.
-  “*format::file:entry*”: combination form of file's organization format, name, and index ID.
-  “*database:entry*”: combination form of database name and index ID.
-  “*database*”: database name.
-  “@*file*”: read each line in the file as an input sequence.

*Seqret* can recognize the form “*format::file*.” For example, if we have a sequence file *file.seq* of the fasta type, we can express it as “*fasta::file.seq*.”

In addition, *seqret* still contains two senior parameters: -feature shows the characteristics information of the sequence applied, and *-firstonly* indicates that the program terminates after reading a sequence from the sequence file.

### 2.2. ORF Search Tools *Getorf*

*Getorf* is used to find the *ORF* in the known RNA sequence and translate the obtained polypeptides.

The parameters of *getorf* are as follows.

 (1) Input sequence file: nucleic acid sequence that corresponds with the USA formats.
 (2) Output sequence file: gene sequence file that includes the *orf* search results.
 (3) Senior options: -*circular* indicates whether the gene sequence is a ring, -*reverse* indicates whether to find ORF in the gene's completely reverse sequence, and -*flanking * indicates choosing a chain of branched gene sequence between the beginning and ending codons.
 (4) Additional limited options: -*minsize*, -*maxsize*, -*find*, and -*table*.
   (a) -*minsize* indicates that the program needs to search a peptide sequence with the length not less than minisize.
   (b) -*maxsize* indicates that the program needs to search a peptide sequence with the length not more than maxisize.
   (c) -*find* is followed with digital options. The meaning of specific number is described in Table 1.
   (d) -*table* is followed with menu number from 0 to 23 to represent the organism's types. Here we do not describe the interpretation of specific number in detail.

### 2.3. Molecular Clock Based Maximum Likelihood Estimation Tools *Proml*

*Proml* is mainly used to construct amino acid sequence tree based on molecular clock maximum likelihood estimation. *Proml* has one input parameter which is a sequence file with *phy* format, and the sequence file includes numbers of amino acid sequences. Although the input parameter of *Proml* is very simple, but it has complex parameters settings, here we leave out the specific set options list.

## 3. Framework of Bioinformatics Software's Encapsulation and Publication

**Figure 1:**
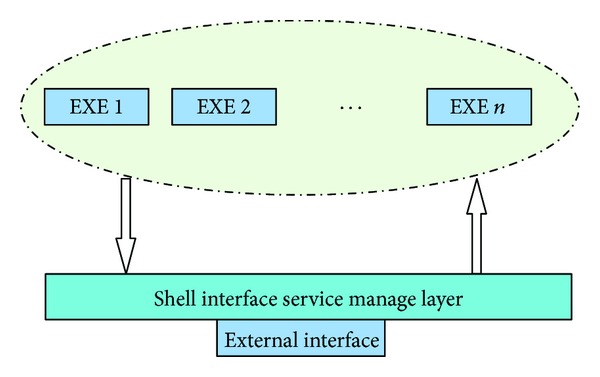
Encapsulation of the bioinformatics software.

Firstly, according to the characteristics of bioinformatics software, we extract a unified interface to make it easy to integrate a lot of bioinformatics software. Thus, we encapsulate a layer of shell over the bioinformatics software, just as described in Figure 1. The shell program exposes a simple interface to the outside, thus making it convenient to publish the service.

Secondly, we embed the encapsulated bioinformatics software in the compiled GRAM service [9]; GRAM called this application and provided bioinformatics software's service to the outside [10]. The publishing part usually uses the web service. In the publishing interface, we premise that the users already know the existence of GRAM service, so that we can use the API that is provided by GRAM service to call the application sources that the GRAM contains; then bioinformatics software's publishing is realized.

**Figure 2:**
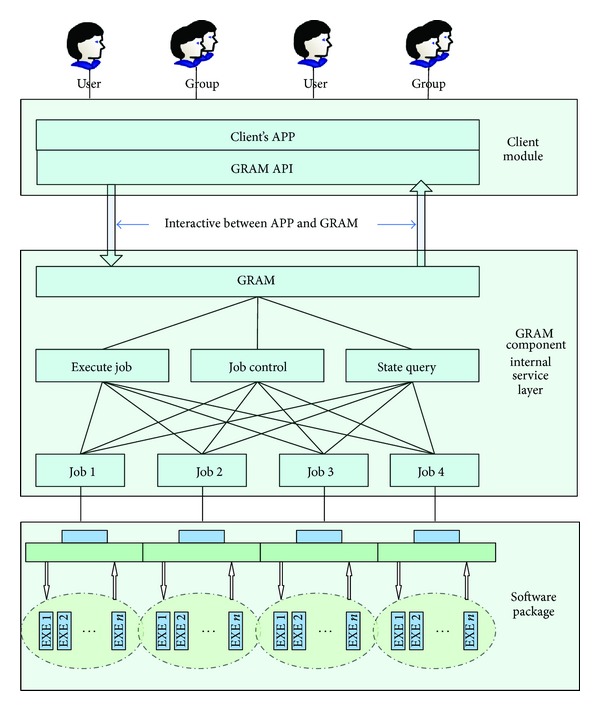
Framework of bioinformatics software's encapsulation and publication.

Therefore, we form the framework of bioinformatics software's encapsulation and publishing, as Figure 2 shows.

As shown in Figure 2, in the upper level of our encapsulated software, GRAM encapsulates another layer, namely, the GRAM component internal service layer. The software is published with GRAM service's publication. During the publication, we use the API that is provided by GRAM to publish the bioinformatics software's function to the users and finally realize the bioinformatics software's publication.

### 3.1. Software Encapsulation on Windows Platform

As the software that will be encapsulated is all executable files, we create process to execute the exe files and deal with the interactive process by redirecting the standard input and output. During the encapsulation process, the main part is the application of redirection technology.

The specific redirecting process is as follows: we assume that there are two anonymous pipelines, two one-way pipelines: pipeline A and pipeline B; each pipeline has one input terminal and one output terminal. 

First step: if we want to execute a command, we need to put this command to the execution file's process. We use *hStdInput* to stand for standard input; it is originally responsible for receiving the user's input from the keyboard; here we hang it up on pipeline A's output terminal and make it responsible for receiving pipeline A's output data.

Second step: now we have connected pipeline A's output terminal to the input terminal of execution file's process; namely, pipeline A's output terminal is execution file's input terminal, so that, if we write a command to pipeline A's input terminal, the execution file can get our command through pipeline A.

Third step: as known, it is impossible for pipeline A to receive the output data of execution file, so we need another pipeline, pipeline B, to receive it. We use *hStdOutput* to stand for standard output, it is originally sent to the screen, and here we hang it up on pipeline B's input terminal and make pipeline B responsible for receiving exe file's output data. What is more, *hStdError* is standard error output, it is also originally sent to the screen, and we hang it up on pipeline B's input terminal too.

Forth step: now pipeline B's input terminal is connected to the output terminal of execution file's process, so that pipeline B's output terminal is bioinformatics software's output terminal; software can receive data from this terminal and send it to the users or use it for further judgment.

### 3.2. Software Encapsulation on Linux Platform

The first step: create two pipelines in the parent process: pipeline 0 and pipeline 1; each pipeline has two terminals, respectively, for reading and writing. For each pipeline, two file descriptors will be generated: one is used to read data from specific file and the other is used to write data to the specific file.

**Figure 3:**
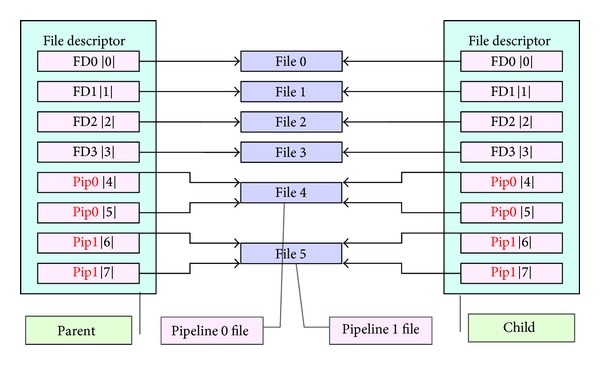
Mapping of parent and child process's file descriptor table.

The second step: call *fork*() to create a new child process. So there are two pipelines for both the parent and child processes, including four descriptors, respectively, for reading and writing two specific files. The two specific files are, respectively, indicated by the four descriptors of the parent and child processes. The general situation is shown in Figure 3.

**Figure 4:**
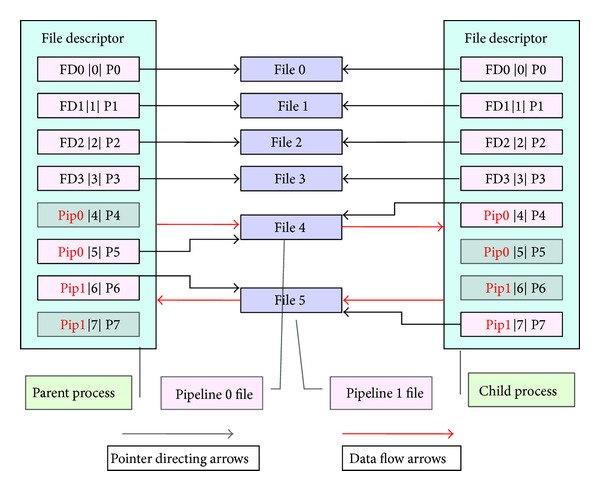
Piping communication schemes of the parent and child processes.

The third step: for pipeline 0 and pipeline 1, respectively, turn off one pipeline's reading terminal and the other pipeline's writing terminal between the parent and child processes. For example, turn off pipeline 0's reading terminal and pipeline 1's writing terminal in the parent process; accordingly, turn off pipeline 0's writing terminal, and pipeline 1's reading terminal in the child process. Thus, the parent process can write data to pipeline 0's file through its writing terminal, and then the child process can read its parent process's data from pipeline 0's reading terminal. And similarly, the child process's feedback information can be written to pipeline 1, and then the parent process can receive the child process's information. So the communication between the child process and parent process is realized. The specific procedure is shown in Figure 4.

The forth step: we need to redirect the standard input/output of the child process. Here, we need to call function *dup2*() in the child process, change the file that the child process's standard input/output indicates by *dup2*(), and finally finish the redirecting of the standard input/output.

### 3.3. Bioinformatics Software's Publication

After encapsulating bioinformatics software, we should publish it next. Firstly, we further encapsulate the software with a GRAM service provided by Globus. Secondly, we publish the GRAM service. By these two steps the bioinformatics software is published.

We compile a client application by using GRAM component's API, and the users can call the bioinformatics software through this application. Next, we introduce the client's realization in detail.

Firstly, we take a look at the GRAM API that Globus project team [11] provides to us. Globus project team publishes an encapsulation named *org.globus.gram*, and this encapsulation realizes all the necessary API functions that are needed when calling the GRAM function. We mainly call the functions of class *Gram* and *GramJobListener* to complete the client program and realize the process that submitting remote services through the GRAM components.

Secondly, we consider the specific process to submit services. When the users call GRAM services' specific application, the main task is to finish the compiling of the resource description file, namely, forming an RSL file. RSL is a cloud resource description language based on XML language. RSL defines various kinds of labels to describe the resource, methods, and details of the calling process.

The users' tasks can be divided into two kinds, namely, single job task and multiple jobs task. For example, the RSL file is a description file of single job task. A single job task contains only one job, while a multiple jobs task contains numbers of jobs.

The submitting modes of user's tasks can be also divided into two kinds, namely, batch mode and no batch mode. In batch mode, the application program will be blocked after the user submits tasks and return after the tasks are completed and the results are returned, while, in no batch mode, the application program returns after the user submits tasks, so that the user can continue to deal with other tasks. If the user wants to observe the specific conditions of the submitted task, he can query the task's status by calling the task examination management interface provided by GRAM.

**Figure 5:**
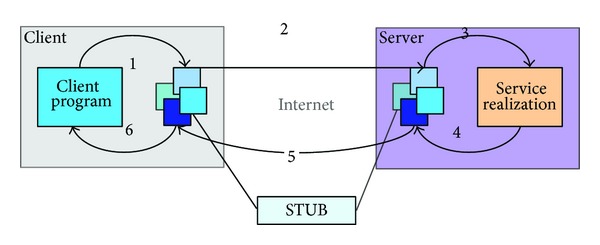
Description of the communication between client and server.

The XML documents form the standards and principles of the communication between applications. We united describe the communication content between the client and the server through XML documents, and the specific communication form is described in Figure 5.

As shown in Figure 5, line 1 shows that client program sends the information that the user requires to the stub module called by the client. Line 2 shows that the stub module encapsulates the information into standard format according to the provision way and measure and sends the encapsulated information to the server stub module. Line 3 shows that the server stub module analyzes the received information and gets the information that the user demands and then sends this information to the service realization program, so that the program can deal with the user's requirements. Line 4 shows that the service realization program sends the processed information back to the stub module. Line 5 shows that the server stub module encapsulates the processed information and sends it to the client stub module. Line 6 shows that the client stub module analyzes the information and feeds it back to the client program.

## 4. Experiments

### 4.1. Encapsulation Interface Test on Windows Platform

First of all, we test the correctness of the bioinformatics software on Windows platform. We encapsulate three application programs, namely, *seqret.exe*, *getorf.exe*, and *proml.exe*. As described in the design, the encapsulation interface has three required parameters, including the file name, the input file, and the output file, and two optional domains, namely, the static input parameter and the dynamic input parameter.

**Figure 6:**
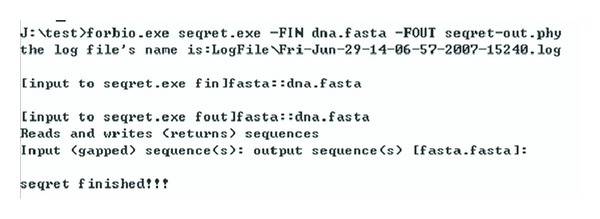
Schematic diagram of calling *seqret's* encapsulation Windows platform.

Firstly, we test the correctness of *seqret's* encapsulation with the three required parameters; the result is shown in Figure 6. Obviously, the test is successful.

**Figure 7:**
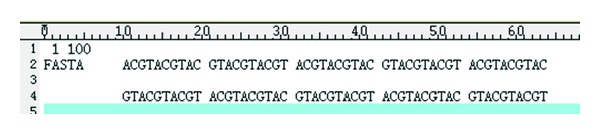
Result of the sequence's transformation.

After calling *seqret's* encapsulation, the corresponding result file *seqret-out.phy* is generated in the directory from which the program runs, and the corresponding log file is also generated in the LogFile folder. The result is shown in Figure 7.

Next, we test *proml's* encapsulation. As *proml* is an application program on Windows platform, so we only test its encapsulation on Windows platform. *Proml* chooses the interactive parameters to communicate with the users. In our test, we choose the parameter –I and import three interactive parameters: u, 5, and y.

**Figure 8:**
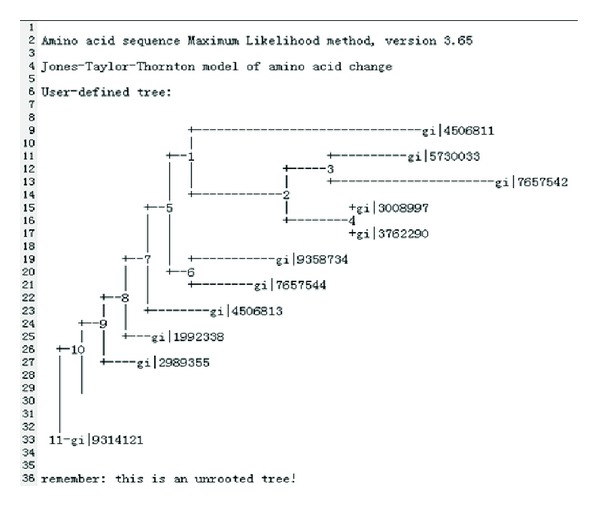
Result of file of *proml. *

The test results are shown in Figure 8.

The log files record the implementation details of this software's calling process successfully, and its format is the same to the log file of *seqret*; here we leave out the details of the log file.

### 4.2. GT's Local Task Submitting Test on Linux Platform

**Figure 9:**
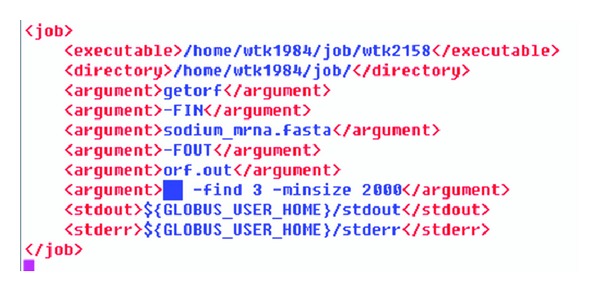
Task description file.

The operating system we use is Red Hat Enterprise Linux Advanced Server 4 [12], and the GT's version is Globus Toolkit 4.0.2 [13]. In the submitting test, the description file is shown in Figure 9.

Firstly, we submit the task by using the command globusrun-ws of GT's command line tool. Before the submit, we need to generate an agent with the command grid-proxy-init firstly; this is because the agent can help do some necessary operation when the GRAM calls other remote file transfer tasks, and the user's certification is needed to be identified. Therefore, the user's certification is the precondition of GRAM components' application.

Next, we take the task description file shown in Figure 9 as one of the parameters we input in the command line to submit our task. In the task description file, we call *getorf* and input parameters *sodium_mrna.fasta*, *orf.out*, and -find 3-minsize 2000 as static parameters.

The result file and log file are as shown in Box 1: as the red front shows, the result file and log file are generated successfully. We check the records in the two files, and they are both correct.

### 4.3. Client Remote Calling Test

We divide the test types of this part into five kinds: 0 cloud node and its service query, 1 remote submitting of nonbatch mode and single job task, 2 remote submitting of batch mode and single job task, 3 remote submitting of batch mode and multiple jobs task, and 4 query of the status of the task that is submitted with batch mode. Then we show the task submitting test of types 0, 1, 3, and 4 in detail.

**Figure 10:**
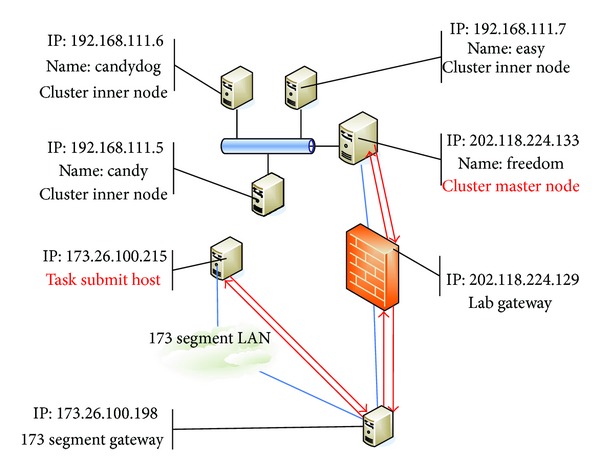
Frame of the network on which the test is conducted.

Before the test, we describe the logic of the network on which the test is conducted. Its frame is shown in Figure 10.

The red font shows the task submitting node and the cluster master node, the place where our task is processed. Our encapsulated software is stored in the master node. The red arrows indicate the flow of the information.

**Figure 11:**
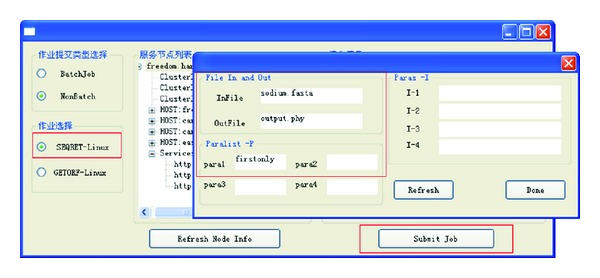
Submitting of no batch mode and single job task.

We test the task's submitting with test type 1, call *seqret* job, and process the gene sequence transfer job.  Figure 11 shows the process of this test.

As Figure 11 shows, we can choose the task and their modes in the most left red box, set the task's parameters through the popup dialog box, and then submit the task by Submit Job button. As the submitted task is nonbatch, the application program blocks after the submitting until the remote execution is finished, and the completion signal is returned.

Next, we login the server and check the result file and the log file; they are both correct. Here, we leave out the details of the inspection.

Finally, we test the task's submitting with test type 3. We check the task's status by calling the query function.

**Figure 12:**
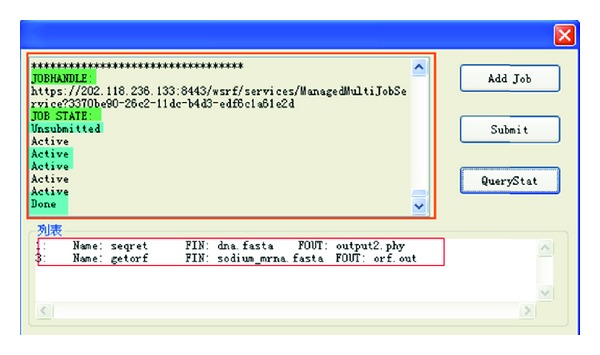
Query of the task's status.

As shown in Figure 12, the list in the bottom shows the tasks' name and parameters in detail, and the upper windows shows the results of the query; from it we can see two items: the job handle and the job state. We can see that our submitted task experiences the process of *Unsubmitted*->*Active*->*Done*. 

## 5. Conclusion

According to the secure encapsulation and publishing of bioinformatics software, this paper introduces the methods of encapsulating and publishing the existing services in the cloud environment and realizes a prototype system to publish bioinformatics software in the grid and cloud computing environment.

In the publishing part, the main process is the analysis of the application software's business and data flow. During the analysis, according to the interaction between processes, we use the communication mechanism of the processes to simulate the man-machine interaction by the application of the pipeline's redirection technology. Finally, according to the results of the analysis, we summarize the characteristics of bioinformatics software's external interface and make the interface simple and universal.

We use the services provided by cloud development tools in the publishing process, write interface processing program for specific application programs, and publish it with corresponding publishing mechanism. Finally, we combine the remote calling of bioinformatics software with cloud environment and form the prototype system of the biological cloud.

## Figures and Tables

**Box 1:**
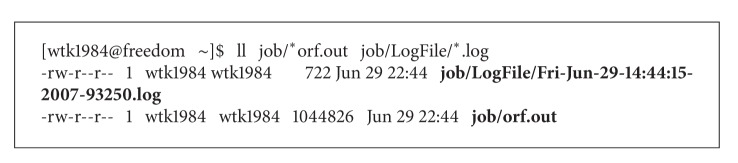


**Table 1:** Interpretation of -*find's* digital options.

Digital option	Interpretation
0	Translate the *orf* between adjacent end codons
1	Output the *orf* between the beginning and ending codons
2	The nuclear sequences between end codons
3	The nuclear sequences between the beginning and end codons
4	The nucleosides side linking with the beginning codon
5	The nucleosides side linking with the start end codon
6	The nucleosides side linking with the terminal end codon
